# Reply to Dr. Hughes

**Published:** 1888-01

**Authors:** J. T. Kent


### REPLY TO DR. HUGHES.

MESSRS. EDITORS: The foot-note on page 400 of your November (1887) issue, leads me to make the following remarks:

While treating a rheumatic subject for slight pains, I was hastily called to her bedside. It was about ten P. M. That morning I had given her Bryonia$^{1m}$ (J.). She greeted me with the following words: "Doctor, the first dose of your medicine gave me pain in the side of my head and temple; every dose increased the pain, until now I cannot stand it. Every time I turn on the right side the pain goes to that side; if I turn on the other side the pain is there."

Thus far *Puls.* and Phos-acid were the only remedies known to me for *pain in the head going to the side lain on.*

Is this Bry., or is there a new feature coming up? The Bry. was stopped and the pains soon stopped. In the morning I satisfied my curiosity by calling at the house, and found her well. She has had no more rheumatism and never had such a headache before or since. Several times have I given Bry. when nearer the general symptoms than Puls. or Phos-ac., for pain going to the side lain on, and have thereby verified this symptom as belonging to the pathogenesis of Bryonia. It would be unwise in me to report this symptom to Dr. Hughes as a pathogenetic symptom. Why? It would be rejected as coming from the 1m potency, "*not reliable.*" Also, must I refuse to report thousands of other symptoms procured in like manner and standing the test of verification in the hands of hundreds of able and faithful men. This "empirical" practice is not based upon the "*Cyclopœdia,*" (?) and why is a Cyclopædia thus entitled to a name that omits the best symptoms to practice on? Time will show forth the merit of the great *opus.* It must stand or fall for itself, and so will the methods based upon one corner of the philosophy of Homœopathy, and rejecting the means whereby the law can be made universal, I mean plainly and simply attenuations above the 12th. I have but the highest regard for Dr. Hughes as a professional gentleman, but must openly protest against the rules for compiling pathogenetic symptoms—for the *Encyclopædia of Drug Pathogenesy*—only the crudest image of the drug being observed. If this one-sided drug image can furnish a basis for correct prescribing it remains to be observed in the distant future, while the evidence of the past stands out in bold condemnation.

The supporters of the crude system have never exhibited anything but a desire to create a very poor materia medica, poor enough to fit the slovenly methods of their practice. The crudest medicines and the crudest methods have marched by the side of grumbling materia medica men.

Does it not seem rather singular that these sticklers for crude drugs are mostly alternationists, Quinine paliationists, cathartic givers, local applicationists, and so on? They acknowledge their own inability to use the materia medica to cure the sick, and do not believe that any one else can use it for that purpose. Will they do better after the *Cyclopœdia* is handed to them? If not, of what good is this great work? It is to be hoped that they will greatly improve, become more scientific (!) and that the dear people will be the ones benefited. As to my "published lectures," I have but a few words to say. They must be quite imperfect, as they are off-hand, class-room talks, and mostly go to the press with scarcely a glance at the reporter's notes; at best they are only journal reading, but with all of these shortcomings they go into the race for the clinical test, to be measured by the first paragraph of the *Organon* of Samuel Hahnemann. "The sole duty of the physician is to restore health to the sick." The objects of the *Cyclopœdia* seem not to sustain this paragraph, but to make compilation of bobtailed drug-effects over-thronged into a chaotic jumble. Individualization would be quite impossible if compelled to rely upon pathogenetic symptoms as found in this work, but it is named a "*cyclopœdia*," and therefore presumed to contain the complete knowledge of the provings on the healthy man. But it is not a *cyclopœdia*. Then what is it? It is a garbled toxicology. made to show the strength of the majority and the remedy most certainly will be administered by the hand of time when the dusty unworn pages are found upon unfrequented shelves in the library of lazy doctors and in the dingy corners of second-hand book-stands. As a toxicology it would be of service but as a pathogenesy it is a travesty.

J. T. KENT.

